# Challenges in achieving more sustainable anaesthesia practices: a narrative review of waste reduction

**DOI:** 10.1016/j.bjao.2025.100406

**Published:** 2025-04-22

**Authors:** Erlend J. Skraastad, Ofelia L. Elvir-Lazo, Paul F. White, David Chernobylsky, Ravina Brring, Roya Yumul

**Affiliations:** 1 Clinic of Anesthesia and Intensive Care, St. Olavs Hospital, Trondheim University Hospital, Trondheim, Norway; 2 Department of Circulation and Medical Imaging, Faculty of Medicine and Health Sciences, Norwegian University of Science and Technology, Trondheim, Norway; 3 Department of Anesthesiology, Cedars-Sinai Medical Center, Los Angeles, CA, USA; 4 White Mountain Institute, The Sea Ranch, CA, USA; 5 Department of Anesthesiology, UC Davis Medical Center, Sacramento, CA, USA; 6 David Geffen School of Medicine at UCLA, University of California, Los Angeles, CA, USA

**Keywords:** anaesthetic gas, anaesthetic waste, climate change, hospital waste, operating room waste, recycling, sustainability

## Abstract

Anaesthesia and operating room waste contribute to climate change and environmental pollution. Although professional bodies involving anaesthesiologists and surgeons have developed guidelines for supporting environmentally conscious health practices, practitioners face difficulties implementing these protocols in hospitals. This narrative review explores the current literature concerning the environmental impact of anaesthesia and operating room waste. We also discuss obstacles practitioners face in implementing initiatives to decrease anaesthetic and surgical waste. A literature review of peer-reviewed publications was conducted across PubMed, MEDLINE, Google Scholar, and Cochrane Databases to identify relevant articles published from 1980 to 2025. A search of recent anaesthesia, surgery, and medicine journals provided additional data. Around 2% of urban solid waste is medical waste, and healthcare accounts for 5% of global greenhouse gas emissions. Scientific organisations have clearly defined guidelines and recommendations to reduce environmental impact. Barriers to implementing existing recommendations include the lack of proper facilities for recycling waste materials, provider workload concerns, lack of hospital leadership, lack of specific targets and accountability measures, insufficient education, and overall resistance to change. Anaesthetic and solid operating room waste pose significant global health concerns, necessitating a collective effort towards sustainability. Anaesthesia and operating room professionals must recognise their responsibility for promoting environmentally friendly practices. Hospital leadership is pivotal to creating a supportive framework. Hospitals and healthcare systems should be required to adhere to specific targets and accountability measures. Meaningful change requires collaboration with a wide range of stakeholders, including politicians, patients, the medical industry, and scientific organisations.

The World Health Organization declares that the ongoing climate change presents a fundamental threat to human health and challenges healthcare services around the world to do more to curtail this growing problem.^1^^,^^2^ The global healthcare sector has a large climate footprint, accounting for 4–8% of global net greenhouse gas (GHG) emissions and contributes to growing healthcare solid waste problem.^3^ Surgery and perioperative services are high-energy and resource-intensive areas that generate approximately 30% of all solid hospital waste and the majority of the hospital's carbon footprint.^4^, ^5^, ^6^

There is a growing recognition worldwide of the healthcare waste problem and the healthcare systems' contribution to GHGs, leading to a call to promote actions that reduce waste and carbon emissions.^7^, ^8^, ^9^ This recognition is based on thorough and recognised reports from global organisations such as the annual *Lancet Countdown on health and climate change*, and *Health care's climate footprint* from Health Care Without Harm.^3^^,^^10^ In March 2023, desflurane was withdrawn from the National Health Service (NHS) in Scotland and from the NHS in England in March 2024.^9^ The European Commission has adopted a ban in EU member states against fluorinated GHGs from 1 January 2026. This prohibits the use of desflurane as an inhalation anaesthetic unless it is strictly required for medical reasons and no other anaesthetic can be used on medical grounds. Further, healthcare facilities must maintain documentation of this medical justification and provide it to the competent authorities upon request.^11^ This is an example of external politics-driven decisions with implications for daily anaesthesiology-related work.^12^

More research in perioperative sustainability is needed to provide evidence-based recommendations. Until then, we have to rely on consensus expert opinion such as the statement from the World Federation of Societies of Anaesthesiologists and the European Society of Anaesthesiology and Intensive Care (ESAIC) consensus document on sustainability.^13^^,^^14^ The American Association of Anesthesiologists, the European Society of Anaesthesiology, the UK Royal College of Surgeons, and the World Federation of Societies of Anaesthesiologists have all presented guidelines to mitigate GHG emissions, reduce waste, and improve sustainability.^13^^,^^15^, ^16^, ^17^

In the 1980s–90s, anaesthesia and operating room (OR) waste surged because of concerns about blood-borne diseases (e.g. human immunodeficiency virus and hepatitis) and iatrogenic transmitted prion diseases (e.g. Creutzfeldt–Jakob disease), with a shift to single-patient use medical equipment (e.g. airway equipment and surgical instruments), surgical gowns, and drug supplies (replacing multidose bottles with single-dose vials).^18^ Since then, anaesthesia and OR waste has continued to increase at an exponential rate, with data linking waste to global pollution.^19^^,^^20^ Apart from the environmental impact, these wasteful practices produce a huge financial burden for hospitals.^19^

There is also growing concern about the potential direct effects of certain plastics, particularly in paediatric health care. Young children and unborn children cannot metabolise chemicals like adults. Although little is known about the direct effects of plastics on human health, there are worries about phthalate exposure from the complex materials used in paediatric care, such as incubators, respiratory circuits, i.v. equipment, and enteral feeding supplies.^21^ Premature newborns and infants are especially vulnerable to the effects of phthalates because of their developing reproductive systems and significantly higher relative phthalate intake.

Climate change impacts healthcare workers and systems in two ways. In addition to adaptation to climate change and its consequences for healthcare professionals, including the emergence of new diseases or the increase in pre-existing ones, mitigation techniques are also required.

The literature for this narrative review is generally based on high-income countries. Despite this limitation, many of the measures described apply also to low- and middle-income countries. According to Health Care Without Harm, Australia, Canada, Switzerland, and the USA have the highest healthcare emissions per capita. They are followed by a number of European countries.^3^ There is generally a correlation between the climate footprint of healthcare and the overall national emissions pattern.

This narrative review focuses on methods and measures to decrease the environmental impact of therapeutic practice, with two objectives: primarily, to review the literature currently available on the effects of anaesthesia and OR waste on the environment; and secondarily, to explore the challenges professionals encounter when putting plans to reduce surgical and anaesthetic waste into action.

## Methods

A literature review of peer-reviewed publications was conducted across PubMed, MEDLINE, Google Scholar, and a Cochrane Databases to identify relevant articles published in English from January 1980 to January 2025, using search terms including but not limited to: ‘operating room waste and environmental pollution’, ‘anaesthesia waste and pollution’, ‘anaesthesia waste and climate change’, ‘anaesthesia waste and global warming’, ‘disposable operating room waste’, ‘surgical gown and drapes waste’, and ‘disposable surgical instrument waste’.

Based on the initial literature search in the databases, the abstracts and titles were screened for relevance by the authors and included or excluded accordingly. The authors assessed whether the articles addressed the environmental effects of anaesthesia and surgical waste in preoperative, intraoperative, postoperative, and surgical treatment practices.

## Results

The initial step in the literature search process revealed a lack of studies and data that can provide high-quality evidence in this field. Additional data were obtained through searches of recent anaesthesia, surgery, and alternative medicine journals. This was done by manually searching the references, the citations of the included articles, or both to identify other relevant material, including ‘grey’ literature.

Few comparative data were found; most were opinion and consensus papers, white papers, guidelines, and case studies. The topic of anaesthesia waste is wide, so there is also a great variety in the studies that have been conducted. For this reason, we conducted a narrative review to provide an overview of the topic and identify areas of interest for future research. We choose to present and discuss the findings from the extended literature search process together with our interpretation.

This narrative review examines anaesthesia and OR waste, available methods for waste mitigation, and proposed pollution reduction strategies. In addition, we also address common obstacles to more environmentally conscious anaesthesia practices.

## Discussion

### Environmental impact of operating room waste

Specific types of anaesthesia and perioperative OR waste include inhaled anaesthetic vapours, i.v. drugs, and OR disposable equipment and supplies. The short- and long-term environmental health impacts of OR waste can vary significantly depending on the type of waste materials. To our knowledge, no controlled studies have been conducted to explore this critical issue. To assess the overall global damage, it is crucial to identify the processes of waste production and elimination. In Table 1, we have summarised the environmental impact of each type of OR-related waste material.

**Table 1:** Type of anaesthesia and operating room (OR) waste and environmental impact and barriers *vs* strategies to overcome barriers.

Type of anaesthesia and OR waste	Environmental impact
Inhaled anaesthesia	Heat trapping as a result of Global Warming Potentials released into the atmosphere, leading to increased rates of climate change and bio-destruction
I.V. anaesthesia	Buildup of toxins with high Persistence–Bioaccumulation–Toxicity scores in aquatic environments and drinking water, increasing likelihood of bio-destruction
OR solid and biohazard waste	- Waste incineration contributes to air pollution and greenhouse gases to be released into the atmosphere - Landfill sites leaking toxins into nearby and distant residential areas, increasing the likelihood of spreading disease and increasing mortality rates
**Barrier**	**Strategy to overcome barrier**
Lack of leadership	- Hospital management: prioritise greening policies on the agenda - Organisation culture: improvements in organisation culture via re-education of staff - Surgeons: choice of reusable *vs* disposable surgical equipment - Ancillary OR staff: emphasis on teamwork among staff members to facilitate lasting changes (team leader recommended for transparency) - Education programmes regarding environmental impacts of various inhaled anaesthetics and i.v. anaesthetics - Education on best medical practices resulting in reduced anaesthesia and OR waste production
Infection risk perceptions	- Education regarding cleanliness and sterility as effected by disposable *vs* reusable equipment - Education regarding proper waste management techniques
Patient safety misconceptions	- Proper education programmes relating the effects of sustainability practices on maintaining patient safety - Education on when sustainability measures may affect patient safety in order to elucidate situations that balance environmental safety with patient safety
Workload concerns	- Making greening initiatives an ‘easier alternative’ - Waste bins conveniently located and marked - Rewarding staff for implementing sustainable practice measures
Staff attitudes	Education and mandatory retraining on best practices as they relate to anaesthesia, OR management, and surgical practices

### Inhaled anaesthetic waste

The most used inhaled anaesthetics are sevoflurane, desflurane, nitrous oxide (N_2_O), and isoflurane.^22^ These are all potent GHGs, and isoflurane and N_2_O are also ozone-depleting agents.^23^^,^^24^ Anaesthetic gases are responsible for 5% of the total carbon footprint of the UK's NHS acute organisations and represent 50% of all healthcare gas emissions.^25^ These numbers are similar to those reported from healthcare organisations in the USA.^26^

Volatile anaesthetics undergo little metabolism in the body and ≥95% are exhaled unchanged into the atmosphere. In the atmosphere, sevoflurane has a lifetime of 1–5 yr before it disintegrates, isoflurane 3–6 yr, desflurane 9–21 yr, and N_2_O 115 yr.^23^ The Global Warming Potential (GWP_100_) is a measure of the amount of heat a given gas traps in the atmosphere over 100 yr compared with a similar mass of carbon dioxide (CO_2_).^27^ The updated GWP_100_ of sevoflurane is 144, N_2_O 298, isoflurane 539, and desflurane 2590.^28^ However, the timeframe of GWP used is of interest as the GWPs based on a shorter timeframe will be larger for gases with lifetimes shorter than that of CO_2_. All anaesthetic gases have a shorter atmospheric lifetime than CO_2_, which means that use of a 20-yr GWP seems to be more accurate in this context than a 100-yr GWP. In that case, GWP_20_ of sevoflurane would be 508, isoflurane 1800, N_2_O 273, and desflurane 6810.^29^

To illustrate the impact of each gas on the environment, investigators converted 1 h of anaesthesia to the distance travelled by an average car. For example, 1 h of 1 minimum alveolar concentration (MAC) sevoflurane is similar to driving 6.5 km, 1 h of 1 MAC isoflurane is equivalent to driving 14 km, 1 h of 60% N_2_O use is equivalent to driving 95 km, and 1 h of 1 MAC desflurane use is equivalent to driving 320 km.^30^

### Volatile anaesthetic gas capture technology

Scavenging systems remove used anaesthetic gases from the anaesthesia breathing circuit and vent these gases to the outdoors to prevent unhealthy working conditions in the OR. Technology to capture and recycle or destroy used volatile anaesthetic gases has recently been introduced. Current volatile gas capture technology (VCT) uses an activated charcoal or metal-organic framework to collect the used anaesthetic gases. Metal-organic frameworks may also have the capability to capture N_2_O unlike conventional scavenging systems. Capture efficiency is important in evaluating VCTs, and two UK-based laboratory studies not involving patients reported approximately 95% capture efficiency.^9^^,^^31^ Recent clinical trial^31^,^32^ reported real-life capture efficiencies of 25% and 51% for two different VCT systems. The optimal solution is for the gases to be recycled and later reused, although to our knowledge it is still only the pharmaceutical authorities of Austria and Germany that have approved the clinical use of recycled sevoflurane.^31^, ^33^, ^34^ It is important to emphasise that this circular economy approach does not apply to desflurane, which is the gas that is of most concern in terms of climate consequences.

Currently, healthcare providers have no financial incentive for capturing waste anaesthetic gases.^35^ Although various commercial devices for VCT exist, there are no independent Life Cycle Assessments (LCAs) of this technology, and it is uncertain if the entire process is more environmentally friendly than conventional scavenging. Regulations and processes regarding waste anaesthetic gases and logistics across borders are still not entirely clear.^14^^,^^36^

While awaiting the arrival of less environmentally toxic anaesthetic gases and more efficient VCTs, current recommendations emphasise the importance of reducing the volume of gases. In the NHS in Scotland, an 82% reduction in volatile gas emissions was achieved over the period of 4 yr, and in Brisbane, Australia, they reduced carbon emissions from volatile anaesthetics by 88% over 5 yr. This is a result of various mitigation measures, such as low-flow anaesthetic gas techniques and switching from desflurane to sevoflurane.^37^^,^^38^ For more than a decade, efforts have been made to manage the fresh gas flow (FGF) in order to reduce environmental contamination.^39^ The clear recommendation from the ESAIC is that FGF should be set to a minimum flow (<0.5 L min^−1^) during the maintenance phase, given no leaks in the breathing circuit system.^14^

For N_2_O, leakage from central pipeline systems is reported from health systems in New Zealand, the UK, and the USA to cause significant losses of N_2_O ranging from 77% to 95%. This leads to recommendations to abandon central piping systems and use portable N_2_O tanks exclusively.^40^, ^41^, ^42^

### Liquid waste

Liquid waste is an important consideration when assessing the environmental impact of i.v. anaesthetic techniques. In addition to propofol, other i.v. anaesthetic drugs, such as barbiturates, etomidate, and ketamine, and several adjunctive drugs, such as dexmedetomidine, droperidol, benzodiazepines, and potent opioid analgesics (e.g. fentanyl and its new analogues), are available. There are limited data regarding the environmental impact of using these drugs in the ORs and ICUs.^43^, ^44^, ^45^, ^46^

Total intravenous anaesthesia (TIVA) and locoregional anaesthesia techniques are potentially more climate friendly alternatives to the inhaled anaesthetics.^23^^,^^47^, ^48^, ^49^, ^50^, ^51^ Even accounting for the plastic syringes and tubing and the electrical power for drug infusion pumps, the GWP of inhaled anaesthetics was found to be four orders of magnitude higher than that of propofol.^52^ Still, propofol-based TIVA techniques can also produce a potential negative impact on the environment from manufacturing processes and wastage (e.g. unused drugs).^53^ This impact is magnified by propofol's market share of anaesthesia pharmaceuticals at ∼34%.^54^

Propofol is environmentally toxic to aquatic environments with a Persistence–Bioaccumulation–Toxicity (PBT) score of 6 on a 9-point scale.^55^^,^^56^ However, the Swedish Drug Agency (FASS) has stated that ‘Propofol has been considered to result in low environmental risk’. This statement is justified using the environmental risk classification, finding the ratio between Predicted Environmental Concentration (PEC) and Predicted No-Effect Concentration (PNEC) which for propofol is 0.14 (i.e. 0.1< PEC/PNEC ≤1).^57^ Propofol metabolism in higher organisms with conjugation and fast renal excretion of the conjugates ensures that only trace amounts of an administered dose will reach the aquatic environment. Studies investigating pharmaceutical pollution in hospital wastewater conclude that propofol is a very minor problem compared with many other molecules.^58^ Still, studies report that propofol wastage ranges from 32–50% of all propofol usage because of improper vial size selection and unpredictable surgery times.^59^ A recent single-centre, prospective study showed that over 2 months, 33% of propofol was wasted and contributed to the largest financial loss of all anaesthetic drugs.^49^ Methods for reducing propofol wastage include reducing the size of propofol vials.^60^ The recommendations from ESAIC's sustainability guidelines are to use 20-ml propofol ampoules, reserve the 50- and 100-ml bottles for infusions, and consider using 2% propofol for longer operations.^14^ There have been no studies assessing the use of single-dose *vs* multidose ampoules of drugs such as propofol, cardiovascular drugs, neuromuscular blocking drugs, and reversal agents as an approach to reducing i.v. drug wastage.^38^^,^^61^

### Solid and biohazard operating room waste

Medical waste is a potential threat not only to employees and patients but also to the surrounding environment and communities. Despite being illegal, medical waste landfilling without pre-treatment is a common method of healthcare waste disposal because it is easy and inexpensive.^62^ In many countries, medical waste is kept in pits or in open landfill sites with municipal waste until burned. The general public and those who handle, collect, and recycle the waste may experience long-term health effects from this.^63^^,^^64^ Additionally, untreated medical waste pollution and surface waste burning contaminate fresh water and soil, harming the environment.^65^ OR waste, including surgical gowns, masks, hats, draping, plastic syringes, tubing, disposable instruments, sharps, and both infectious and biohazardous materials, constitutes 30% of all the solid waste generated by hospitals. A total of 85% of the total amount of waste generated as a result of healthcare activities is non-hazardous, hence potentially recyclable. The remaining 15% is considered hazardous material that may be infectious, toxic, or radioactive.^65^, ^66^, ^67^ However, sharps containers often have improper items deposited into them, with >50% inappropriate materials by weight on average.^68^ Proper sorting and recycling of non-contaminated materials which reduces the biohazardous waste is shown to be effective in reducing not only CO_2_ emission but also the hospital cost of waste management.^69^, ^70^, ^71^

Of note, a significant proportion of OR waste comprises opened but unused supplies and incorrectly sorted potentially recyclable materials.^20^^,^^72^^,^^73^ Unused supplies not only represent material and financial waste but also wastage of the energy and resources used to create them. In a 2017 report across 58 neurosurgical procedures, 13% of the surgical supply cost was from unused disposable materials.^72^ The Stanford University Department of Urology demonstrated that adjusting urological surgeons' preference cards after briefing them about unused disposable items resulted in a significant decrease in OR waste without affecting the quality of patient care.^74^ This simple intervention yielded a 92% reduction in waste. Research into OR waste management across other surgical subspecialties is clearly warranted.

Data from a 1-week waste audit of six ORs in Australia revealed that 25% of the total OR solid waste was anaesthesia-related. Importantly, 58% of anaesthesia waste was shown to be recyclable.^75^ A study comparing reusable and single-use disposable (SUD) laryngoscopes utilised a ‘cradle-to-grave’ LCA and costing methodology to compare SUD metal and plastic laryngoscope handles.^70^ The results indicated that the SUD plastic handle generated nearly 16–18 times more life cycle CO_2_ equivalents than the traditional disinfection of the reusable steel handle.

Considering that most anaesthesia supplies, including syringes, supplies, laryngoscopes, tracheal tubs, breathing circuits, and monitoring electrode pads, are individually packaged in disposable plastic wrappers, the packaging alone from a single surgical case can fill several waste bins.^76^

### Sterilisation techniques and plastics

Sterilisation devices use chemicals that release emissions known to be hazardous air pollutants (e.g. ethyl oxide).^76^ Despite being recognised as a potential mutagen and carcinogen, ethyl oxide remains the gaseous sterilisation agent of choice.^77^^,^^78^ Vaporised hydrogen peroxide is an alternative to ethyl oxide that can be used to reduce hazardous emissions and allow for a reusable alternative which would have a more positive impact on the environment than the expanded use of disposable medical–surgical equipment.^79^

Blue wrap is a No. 5 plastic (i.e. a type of plastic made of polypropylene) and is one of the most common plastics used in OR post-sterilisation equipment wrapping and a major component of gowns and drapes. Blue wrap accounts for 19% of OR waste and is non-biodegradable.^80^ The sterile, uncontaminated, and undamaged blue wrap can be collected for direct recycling. It is demonstrated that, in the OR, surgical wrap can be diverted from the general waste streams to the recycling stream.^81^ This effort has the potential to yield significant reductions to OR waste outputs and to hospital carbon emissions.

The non-biodegradable waste buildup in landfill sites causes damage to both the environment and humans. Plastics are polluting the land, waterways, and oceans, and living organisms are harmed on a daily basis. Both land and sea animals become entangled in plastic materials from packaging and ingest toxic plastic material.^82^ The emission of chemicals from plastics can disrupt physiological processes by functioning as endocrine disruptors, disturbing multiple hormonal mechanisms, and have the potential to cause major health issues for both humans and animals.^82^ Studies have found an association between living near landfill sites and reduced lung function, asthma, ataxia, paralysis, and even lung cancer.^82^ Plastics significantly affect fresh water sources (lakes) and enter the water cycle. There are no fresh water sources free of microplastics on earth.^83^ Microplastic consumption has been linked to gastrointestinal disturbances, endocrine disruption, and transmission of pathogenic bacteria. Inhaling airborne microplastics poses a serious health risk, potentially affecting respiratory and cardiovascular systems. It is less explored whether dermal contact can cause skin irritation and allergic reactions.^84^

### Current barriers to more sustainable practices

#### Leadership and management

A questionnaire-based study sent to 413 anaesthesiology departments in the USA suggested that the top barriers to sustainable anaesthesia practices were ‘lack of leadership’ from the hospital administration and anaesthesia department leadership, ‘perceived infection risk’, ‘provider workload concerns’, and ‘staff attitudes which resist change’.^38^ A national survey involving 2695 anaesthesiologists regarding environmental sustainability reported that the key barriers were ‘lack of OR and hospital leadership’ (64%) and ‘insufficient education’ (63%). Among respondents, 69% were interested in further education; however, only 31% were aware that sustainability programmes existed at their institution.^78^

In the medical literature, the policies of ‘Rs’ of waste management were developed to assist in developing OR waste management strategies and to make the OR more environmental friendly.^20^ The first five Rs then included reduction, reuse, recycling, innovation, and renewable energy. In the current guidelines from ESAIC, the 5R policies focus on waste and consist of: reject, reduce, reuse, recycle, and repair.^14^ The first R, reject, which means to avoid using unnecessary products or devices and avoid waste generation, is probably the most important R, but also the most difficult to achieve.

Protocols for improving waste management have also been developed by the American Society of Anesthesiologists (ASA) Task Force on Environmental Sustainability Committee on Equipment and Facilities.^7^ Information includes proper anaesthetic equipment choices, environmental impact of inhaled anaesthetics, FGF management strategies, the environmental impact of i.v. anaesthetics, and recycling programmes.

Despite the availability of evidence-based protocols and initiatives, there is a lack of significant response from hospital leaders and administrators in implementing these protocols within the hospital system. Leadership is vital because hospitals need guidelines and policies promoting green OR efforts and changing the attitudes of many healthcare workers and hospital administrators.^38^

There are many success stories in which hospital waste and emissions have been reduced, but it is imperative that these experiences do not remain at the level of ‘pilot projects’ and that specific legislation be adopted at the regional, national, and international levels to facilitate the reduction in the environmental footprint of hospitals.^85^

To achieve the warranted global effects by implementing environmental measures locally, acceptance and approval are needed at every level. This is from the local institution management to the national and international politicians and policymakers. Health system leaders must set clear and compelling goals, invest in metrics that can be measured internationally, and embed them into their core business activities.^86^ Specific targets and accountability measures are necessary to force hospitals and healthcare systems to act. Whole-system approaches are seen as more effective when dealing with impact mitigation.^87^

Hospitals have considerable potential for improving sustainability, yet this potential remains untapped.^88^ Taking advantage of this potential is the responsibility of clinicians, healthcare organisations, policymakers, politicians, patients, and suppliers and manufacturers in the supply chain.^89^ To create an environment favourable to the successful transformation of healthcare, policies—at all levels of government and organisation—and regulations at the national level will be crucial.^90^ The NHS has emphasised the importance of incorporating bottom-up data into top-down modelling to improve accuracy.^8^

It is important to emphasise the financial advantages of implementing sustainable practices to make the process more acceptable: one hospital's expenditure on anaesthetic gases was reduced by 58% after cutting desflurane for inhalation anaesthesia, a fact not highlighted in the report.^38^ Hospitals can save a considerable amount of money by reducing their energy and waste management expenses. However, this potential is of no use if the granting authorities do not finance or guarantee the investments required to implement such measures. It is also necessary for the possible financial benefit of process improvement to be apparent at the enterprise level.^91^

#### Education and information

According to a survey conducted by the ASA from 2012, it was reported that although 80% of anaesthesiologists expressed an interest in recycling, 67% indicated a lack of sufficient information regarding intraoperative recycling procedures. Furthermore, the survey revealed a lack of hospital-wide organisational efforts and waste reduction programmes.^92^ This survey suggests that insufficient education and awareness among physicians and hospital staff are significant causes of improper recycling practices.

These findings are congruent with more recent surveys performed in other countries: a Canadian survey from 2019 reported that 98% of the participating anaesthesiologists were willing to recycle OR waste at work, but only 30% did so. Barriers to recycling in the OR included a lack of support from OR and hospital leadership, and inadequate information and education indicated by 64% and 63% of the participants, respectively.^93^ In a Portuguese survey from 2024, 67% of the responding anaesthesiologists attribute ‘great importance’ to the subject of environmental sustainability. As to the greatest barrier to waste separation in the OR, 63% highlighted the issues of ‘inadequate information/education/training’ and 26% the ‘lack of support from hospital/OR in-chief/administration’.^94^

This indicates that anaesthesiologists appear to be ready to incorporate environmental considerations into their practice, and that there is a concern about the impact of hospital waste on the environment. Further, it indicates that significant barriers to recycling have not changed over the past decade. It may be difficult to still consider a lack of information and education as a fundamental barrier to the reduction in hospital waste after over a decade of work in this area, with the implementation of specific targets and worldwide approaches with multiple guidelines available, as cited in this review. However, there may still be issues with the provision of information and education at a local level.

It is timely to consider whether information and training lead to the desired environmental results with a reduction in hospital waste and less emissions. Education reduces the knowledge gap about environmental problems,^95^ and support for climate policies is increased by education that clarifies policy mechanisms, but not that only highlights the effects of climate change.^96^ 10.13039/100014337Furthermore, knowledge regarding climate change is significantly and positively correlated with attitudes toward climate change.^97^ Healthcare personnel consider training and education necessary to equip them for 21st-century planetary health challenges.^98^

A high level of knowledge among health personnel about climate change as a health threat issue does not necessarily affect the ability to promote activities to meet climate change challenges.^99^ Education does not lead directly to action, but it is necessary to involve the health personnel in required changes, hence ‘bridging the gap between climate policy and climate action for healthcare staff’.^100^ As shown in the UK's NHS, which has significantly reduced their carbon emissions by 26% between 1990 and 2019,^8^ information and education are indisputable essential factors in managing changes to a sustainable healthcare system. The need for continuous education is also emphasised by the NHS on the way forward to reach their goal of zero emissions by 2040.^101^

Education is crucial because it can address staff perceptions, patient safety misconceptions, and overwork concerns, and contribute to standardising efficient and environmentally conscious hospital practices. Hospital administrators and healthcare regulatory agencies need to carefully examine the scientific data supporting some of the most wasteful OR practices. Mandatory re-education of hospital administrators, medical staff, nurses, OR technicians, and pharmacists offers the most realistic approach to implementing these common-sense approaches to implementing waste reduction practices in the future.^102^ Practical, evidence-based actions can be taken to reduce the impact of pollution from inhalation anaesthetics. According to guidelines published in *Anaesthesia* 2022,^15^ the majority of Scientific Societies recommend removing desflurane from drug formularies, decommissioning central N_2_O piping, avoiding the use of N_2_O, reducing FGF during anaesthesia to <0.5 L min^−1^, and prioritising TIVA and regional anaesthesia when clinically appropriate.

Greater physician and nursing staff education would help alleviate their concerns regarding infection risks and increased workloads related to promoting proper waste disposal.^38^ The failure to separate infectious waste from clean waste is a costly barrier to recycling hospital waste, and OR waste in particular.^69^ Commercial equipment and technology are now available to sterilise contaminated medical waste, allowing more waste to be recycled.^103^

#### Resistance to change and workload

A review of OR greening initiatives highlighted the simple fact that overall resistance to change is a barrier to improving waste management.^102^ The presence of such resistance is normal and should be expected, recognised, and dealt with appropriately because it can have a significant impact on the success or failure of a proposed change. In response to workload concerns, healthcare providers can be offered a range of meaningful options, allowing them to assess their schedules and choose the commitments they are prepared to take on.^104^ Furthermore, a study has revealed that mitigating time constraints can be achieved through the strategic recruitment and development of volunteers for leadership roles. This approach effectively distributes responsibilities among multiple individuals, fostering a collaborative environment.^105^ By default, effective efforts are durable, and self-interest is a significant factor in perceptions affecting environmental policy support.^96^ Hence, emphasis must be placed on creating working conditions and technical solutions that make it easier to choose environmentally friendly solutions. This includes practical support to make sustainability easier in the workplace (e.g. waste streams, energy supply, logistics, and transport), and embedding sustainability as ‘business as usual’ in healthcare culture and system.^98^ In a report exploring the views of anaesthetic practitioners after the transition from desflurane to TIVA, participants emphasised TIVA's drawbacks, such as increased effort, stricter monitoring needs, operational challenges, and lack of clinical confidence in its application.^106^ Such drawbacks can be a challenge in adopting more environmentally friendly solutions. The same applies to the transition to regional anaesthesia, which requires interest, education, and training in addition to specific equipment.^107^

It must be made easier to choose the most environmentally friendly solution in the OR, and a behavioural change in the OR can be established by introducing a simple recycling programme. Every operating theatre can divert surgical wrap from the general waste stream to the recycling stream, and with the differentiation of non-contaminated material it is possible to reduce the amount of biohazardous waste.^69^^,^^81^

**Figure 1:**
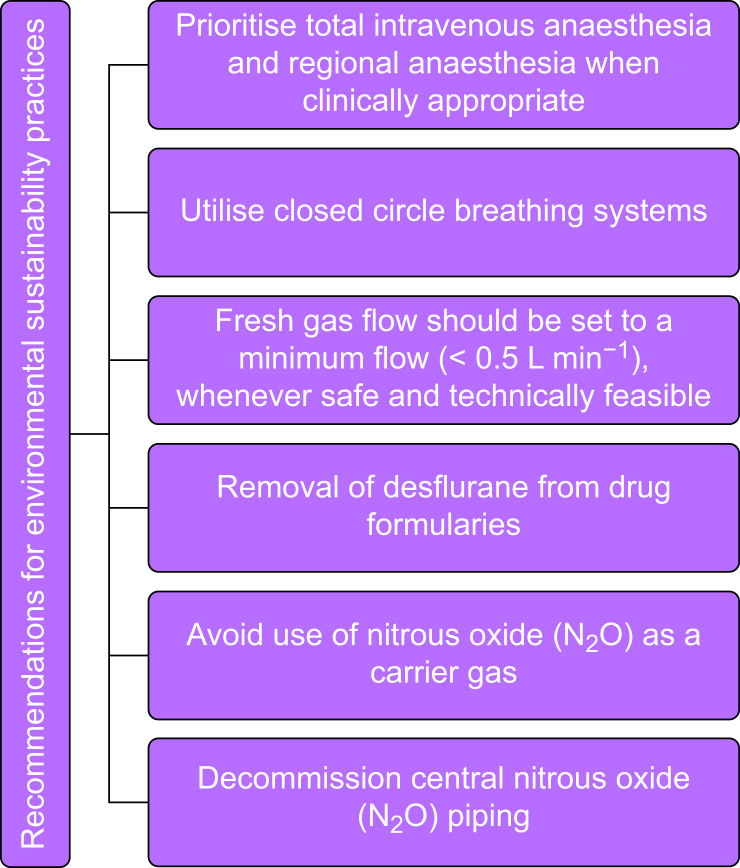
Recommendations for environmental sustainability practices in the operating room.

It can also encourage anaesthesia providers to adopt a different approach to drawing up OR medications. For example, they can stop routinely drawing up emergency drugs.^108^ Open, unused syringes tend to be discarded after a surgical procedure because of the perceived risk of infection leading to unnecessary waste production.^13^ Instead of pre-emptively drawing up medications that will not be used, prefilled syringes for commonly used anaesthetic drugs can be used to reduce cost with minimal, if any, clinical significance.^109^ Additionally, drug ampoules and syringes can be unopened but available for immediate use when preparation is needed.^110^ Individual strategies for dealing with specific barriers are summarised in Table 1.^30^ Fig 1 displays recommendations for environmental sustainability practices at the level of an anaesthesia provider.

## Conclusions

Anaesthesia and OR waste must be reduced. Anaesthesiologists should take a leadership role and work with other hospital employees to improve OR waste management. The necessary changes can only be achieved by acknowledging that each individual anaesthesia professional has an independent responsibility. There is a need for specific targets and accountability measures in order to encourage hospitals and healthcare systems to take action. The potential of hospitals to improve sustainability remains untapped. Achieving this potential is the responsibility of clinicians, healthcare organisations, policymakers, politicians, patients, and suppliers and manufacturers. An understanding of the impact of OR and anaesthesia waste production on the global environment by healthcare providers is essential to implement the changes needed for a more environmentally conscious future. For recommendations and guidelines to be supported, more research is needed on this topic.

## Authors' contributions

Study conceptualisation: DC, OEL, PW, RB, RY

Study design: DC, OEL, PW, RB, RY

Data collection: DC, OEL, PW, RB, RY, ES

Analysis: DC, OEL, PW, RB, RY

Original draft writing: DC, OEL, PW, RB, RY

Supplementation: ES, OEL, PW

Writing, reviewing, and editing the manuscript: ES, OEL, PW

Authorised the final manuscript: all authors

## Funding

Clinic of Anesthesia and Intensive Care, St. Olavs Hospital, Trondheim, Norway; Department of Anesthesiology, Cedars-Sinai Medical Center, Los Angeles, CA, USA; White Mountain Institute, The Sea Ranch, CA, USA.

## Declarations of interest

The authors declare that they have no conflicts of interest.

## References

[1] World Health Organization (2021).

[2] World Health Organization (2024).

[3] Karliner J., Slotterback S., Boyd R., Ashby B., Steele K., Wang J. (2020). Health care’s climate footprint: the health sector contribution and opportunities for action. Eur J Public Health.

[4] MacNeill A., Lillywhite R., Brown C. (2017). The impact of surgery on global climate: a carbon footprinting study of operating theatres in three health systems. Lancet Planet Health.

[5] McGain F., McAlister S., McGavin A., Story D. (2010). The financial and environmental costs of reusable and single-use plastic anaesthetic drug trays. Anaesth Intensive Care.

[6] McGain F., Muret J., Lawson C., Sherman J.D. (2020). Environmental sustainability in anaesthesia and critical care. Br J Anaesth.

[7] Sherman J.D., MacNeill A.J., Biddinger P.D., Ergun O., Salas R.N., Eckelman M.J. (2023). Sustainable and resilient health care in the face of a changing climate. Annu Rev Public Health.

[8] Tennison I., Roschnik S., Ashby B. (2021). Health care's response to climate change: a carbon footprint assessment of the NHS in England. Lancet Planet Health.

[9] Shiralkar S., Field E., Murphy E., Shelton C. (2023). The role of volatile capture technology in desflurane disposal from decommissioned vaporisers. Anaesthesia.

[10] Romanello M., Walawender M., Hsu S.C. (2024). The 2024 report of the Lancet Countdown on health and climate change: facing record-breaking threats from delayed action. Lancet.

[11] Hendrickx J.F.A., Nielsen O.J., De Hert S., De Wolf A.M. (2022). The science behind banning desflurane: a narrative review. Eur J Anaesthesiol.

[12] Gonzalez-Pizarro P., Muret J., Brazzi L. (2023). The green anaesthesia dilemma: to which extent is it important to preserve as many drugs available as possible. Curr Opin Anaesthesiol.

[13] White S.M., Shelton C.L., Gelb A.W. (2022). Principles of environmentally-sustainable anaesthesia: a global consensus statement from the World Federation of Societies of Anaesthesiologists. Anaesthesia.

[14] Gonzalez-Pizarro P., Brazzi L., Koch S. (2024). European Society of Anaesthesiology and Intensive Care consensus document on sustainability: 4 scopes to achieve a more sustainable practice. Eur J Anaesthesiol.

[15] Devlin-Hegedus J.A., McGain F., Harris R.D., Sherman J.D. (2022). Action guidance for addressing pollution from inhalational anaesthetics. Anaesthesia.

[16] Buhre W., De Robertis E., Gonzalez-Pizarro P. (2023). The Glasgow declaration on sustainability in anaesthesiology and intensive care. Eur J Anaesthesiol.

[17] Robb H., Pegna V. (2023). The intercollegiate green theatre checklist. Bull R Coll Surg Engl.

[18] Kagoma Y.K., Stall N., Rubinstein E., Naudie D. (2012). People, planet and profits: the case for greening operating rooms. CMAJ.

[19] Babu M.A., Dalenberg A.K., Goodsell G., Holloway A.B., Belau M.M., Link M.J. (2019). Greening the operating room: results of a scalable initiative to reduce waste and recover supply costs. Neurosurgery.

[20] Guetter C.R., Williams B.J., Slama E. (2018). Greening the operating room. Am J Surg.

[21] Van Vliet ED., Reitano E.M., Chhabra J.S., Bergen G.P., Whyatt R.M. (2011). A review of alternatives to di (2-ethylhexyl) phthalate-containing medical devices in the neonatal intensive care unit. J Perinatol.

[22] Shelton C.L., Sutton R., White S.M. (2020). Desflurane in modern anaesthetic practice: walking on thin ice(caps)?. Br J Anaesth.

[23] Varughese S., Ahmed R. (2021). Environmental and occupational considerations of anesthesia: a narrative review and update. Anesth Analg.

[24] Ravishankara A.R., Daniel J.S., Portmann R.W. (2009). Nitrous oxide (N2O): the dominant ozone-depleting substance emitted in the 21st century. Science.

[25] Einset K., Aitken T., McConnell P. (2023). Sustainability in anaesthesia. Anaesth Intensive Care Med.

[26] Rizan C., Steinbach I., Nicholson R., Lillywhite R., Reed M., Bhutta M.F. (2020). The carbon footprint of surgical operations: a systematic review. Ann Surg.

[27] Özelsel T.J.P., Sondekoppam R.V., Buro K. (2019). The future is now—it’s time to rethink the application of the Global Warming Potential to anesthesia. Can J Anesth.

[28] Sulbaek Andersen M.P., Nielsen O.J., Sherman J.D. (2023). Assessing the potential climate impact of anaesthetic gases. Lancet Planet Health.

[29] Andersen M.P.S., Nielsen O.J., Sherman J.D. (2021). The global warming potentials for anesthetic Gas sevoflurane need significant corrections. Environ Sci Technol.

[30] Hanna M., Bryson G.L. (2019). A long way to go: minimizing the carbon footprint from anesthetic gases. Can J Anaesth.

[31] Hinterberg J., Beffart T., Gabriel A. (2022). Efficiency of inhaled anaesthetic recapture in clinical practice. Br J Anaesth.

[32] Gandhi, Jason (2022). Efficiency of inhaled anaesthetic recapture in clinical practice. Comment on Br J Anaesth.

[33] Vaghela M., Kay R.H., Jones L., Greig P. (2023). Inhalational anaesthetics: an assessment of agent delivery and capture. Anaesthesia.

[34] Kochendörfer I.M., Kienbaum P., Großart W., Rossaint R., Snyder-Ramos S., Grüßer L. (2022). [Environmentally friendly absorption of anesthetic gases: first experiences with a commercial anesthetic gas capture system]. Anaesthesiologie.

[35] Gandhi J., Barker K., Cross S., Goddard A., Vaghela M., Cooper A. (2024). Volatile capture technology in sustainable anaesthetic practice: a narrative review. Anaesthesia.

[36] Müller-Wirtz L.M., Volk T., Meiser A. (2024). Towards sustainability of volatile anaesthetics: capture and beyond. Br J Anaesth.

[37] Wetherell W. (2024). Review of National Health Service England's emergency preparedness, resilience and response annual assurance for 2021-2022. Disaster Med Public Health Prep.

[38] Wyssusek K., Chan K.L., Eames G., Whately Y. (2022). Greenhouse gas reduction in anaesthesia practice: a departmental environmental strategy. BMJ Open Qual.

[39] Feldman J.M. (2012). Managing fresh gas flow to reduce environmental contamination. Anesth Analg.

[40] Chakera A., Pearson F. (2022). Nitrous oxide mitigation, look before you leap. Anaesthesia.

[41] Seglenieks R., Wong A., Pearson F., McGain F. (2022). Discrepancy between procurement and clinical use of nitrous oxide: waste not, want not. Br J Anaesth.

[42] Chakera A., Harrison S., Mitchell J., Oliver C., Ralph M., Shelton C. (2024). The Nitrous Oxide Project: assessment of advocacy and national directives to deliver mitigation of anaesthetic nitrous oxide. Anaesthesia.

[43] Schraag S., Irwin M.G., Wong G.T.C., Lam S.W. (2019). Taking on TIVA: debunking myths and dispelling misunderstandings.

[44] Brodin T., Nordling J., Lagesson A. (2017). Environmental relevant levels of a benzodiazepine (oxazepam) alters important behavioral traits in a common planktivorous fish, (*Rutilus rutilus*). J Toxicol Environ Health A.

[45] Li S.W., Wang Y.H., Lin A.Y. (2017). Ecotoxicological effect of ketamine: evidence of acute, chronic and photolysis toxicity to Daphnia magna. Ecotoxicol Environ Saf.

[46] Vandenberg L.N., Colborn T., Hayes T.B. (2012). Hormones and endocrine-disrupting chemicals: low-dose effects and nonmonotonic dose responses. Endocr Rev.

[47] Kuvadia M., Cummis C.E., Liguori G., Wu C.L. (2020). Green-gional anesthesia: the non-polluting benefits of regional anesthesia to decrease greenhouse gases and attenuate climate change. Reg Anesth Pain Med.

[48] Sherman J.D., Barrick B. (2019). Total intravenous anesthetic versus inhaled anesthetic: pick your poison. Anesth Analg.

[49] Habte M.F., Tegegne B.A., Alemayehu T.Y. (2024). Anesthetics drug wastage and preventive strategies: systematic review. PLoS One.

[50] Kicker J.S., Hill H.S., Matheson C.K. (2018). Better pairing propofol volume with procedural needs: a propofol waste reduction quality improvement project. Hosp Pediatr.

[51] Peker K. (2020). The wastage and economic effects of anaesthetic drugs and consumables in the operating room. Turk J Anaesthesiol Reanim.

[52] Sherman J., Le C., Lamers V., Eckelman M. (2012). Life cycle greenhouse gas emissions of anesthetic drugs. Anesth Analg.

[53] Allen C., Baxter I. (2021). Comparing the environmental impact of inhalational anaesthesia and propofol-based intravenous anaesthesia. Anaesthesia.

[54] Ramirez M.F., Gan T.J. (2023). Total intravenous anesthesia versus inhalation anesthesia: how do outcomes compare?. Curr Opin Anesthesiol.

[55] Sangion A., Gramatica P. (2016). PBT assessment and prioritization of contaminants of emerging concern: pharmaceuticals. Environ Res.

[56] Campbell M., Pierce J. (2015). Atmospheric science, anaesthesia, and the environment. BJA Educ.

[57] Fass - The Swedish Electronic Medicines Compendium, 2025. Available from https://www.fass.se/LIF/product?userType=0&nplId=19870508000066&docType=78.(accessed January 15th 2025)

[58] Waspe J., Orr T. (2023). Environmental risk assessment of propofol in wastewater: a narrative review of regulatory guidelines. Anaesthesia.

[59] Tang M.S.S., McGain F., Bramley D.E., Sheridan N.M., Seglenieks R. (2023). Evaluation of propofol wastage and disposal in routine anesthesia care. Anaesth Intensive Care.

[60] Mankes R.F. (2012). Propofol wastage in anesthesia. Anesth Analg.

[61] Schneider D., Ponto J., Martin E. (2017). Propofol disposal in the anesthesia setting: overcoming barriers. AANA J.

[62] Meleko A., Tesfaye T., Henok A. (2018). Assessment of healthcare waste generation rate and its management system in health centers of Bench Maji Zone. Ethiop J Health Sci.

[63] Khan B.A., Cheng L., Khan A.A., Ahmed H. (2019). Healthcare waste management in Asian developing countries: a mini review. Waste Manag Res.

[64] Ciplak N., Kaskun S. (2015). Healthcare waste management practice in the West Black Sea Region, Turkey: a comparative analysis with the developed and developing countries. J Air Waste Manag Assoc.

[65] Janik-Karpinska E., Brancaleoni R., Niemcewicz M. (2023). Healthcare waste-a serious problem for global health. Healthcare (Basel).

[66] Zikhathile T., Atagana H., Bwapwa J., Sawtell D. (2022). A review of the impact that healthcare risk waste treatment technologies have on the environment. Int J Environ Res Public Health.

[67] Pichler P.P., Jaccard I., Weisz U., Weisz H. (2019). International comparison of health care carbon footprints. Environ Res Lett.

[68] Seidman P.A., Parker B.M. (1998). Sharps disposal in the operating room: current clinical practices and costs. Anesth Analg.

[69] Leone N., Scozzari G., Olandese F. (2024). O.R. Goes GREEN: a first step toward reducing our carbon footprint in the operating room and hospital. Updates Surg.

[70] McGain F., Story D., Hendel S. (2009). An audit of intensive care unit recyclable waste. Anaesthesia.

[71] Assemu D.M., Tafere T.E., Gelaw Y.M., Bantie G.M. (2020). Healthcare waste management practice and associated factors among private and public hospitals of Bahir Dar City Administration. J Environ Public Health.

[72] Zygourakis C.C., Yoon S., Valencia V. (2017). Operating room waste: disposable supply utilization in neurosurgical procedures. J Neurosurg.

[73] Deshpande N.G., Witmer H.D.D., Keceli Ç., Adelman D., Turaga K.K. (2021). Surgical team familiarity and waste generation in the operating room. Am J Surg.

[74] Pesigan P., Chen H., Bajaj A.A., Gill H.S. (2021). Cost savings in urology operating rooms by editing surgeon preference cards. Qual Manag Health Care.

[75] McGain E., Hendel S.A., Story D.A. (2009). An audit of potentially recyclable waste from anaesthetic practice. Anaesth Intensive Care.

[76] Xiao M.Z.X., Abbass S.A.A., Bahrey L., Rubinstein E., Chan V.W.S. (2021). A roadmap for environmental sustainability of plastic use in anesthesia and the perioperative arena. Anesthesiology.

[77] Kwikiriza S., Stewart A.G., Mutahunga B., Dobson A.E., Wilkinson E. (2019). A whole systems approach to hospital waste management in rural Uganda. Front Public Health.

[78] Yazie T.D., Tebeje M.G., Chufa K.A. (2019). Healthcare waste management current status and potential challenges in Ethiopia: a systematic review. BMC Res Notes.

[79] McEvoy B., Rowan N.J. (2019). Terminal sterilization of medical devices using vaporized hydrogen peroxide: a review of current methods and emerging opportunities. J Appl Microbiol.

[80] Albert M.G., Rothkopf D.M. (2015). Operating room waste reduction in plastic and hand surgery. Plast Surg (Oakv).

[81] Rooney D.J., Linehan L., Burke C. (2024). Surgical instrument wrap: a pilot recycling initiative. Ir J Med Sci.

[82] Njoku P.O., Edokpayi J.N., Odiyo J.O. (2019). Health and environmental risks of residents living close to a landfill: a case study of Thohoyandou landfill, Limpopo Province, South Africa. Int J Environ Res Public Health.

[83] Nava V., Chandra S., Aherne J. (2023). Plastic debris in lakes and reservoirs. Nature.

[84] Emenike E.C., Okorie C.J., Ojeyemi T. (2023). From oceans to dinner plates: the impact of microplastics on human health. Heliyon.

[85] Piscitelli P., Karaj S., Miani A. (2023). How healthcare systems negatively impact environmental health? The need for institutional commitment to reduce the ecological footprint of medical services. Epidemiologia (Basel).

[86] Hensher M., McGain F. (2020). Health care sustainability metrics: building a safer, low-carbon health system. Health Aff.

[87] Ryan-Fogarty Y., O'Regan B., Moles R. (2016). Greening healthcare: systematic implementation of environmental programmes in a university teaching hospital. J Clean Prod.

[88] Keller R.L., Muir K., Roth F., Jattke M., Stucki M. (2021). From bandages to buildings: identifying the environmental hotspots of hospitals. J Clean Prod.

[89] Smith C.L., Rojas C., Zurynski Y., Partington A., Braithwaite J. (2024). What Australia must do to create a climate-responsive health system. Intern Med J.

[90] Aboueid S., Beyene M., Nur T. (2023). Barriers and enablers to implementing environmentally sustainable practices in healthcare: a scoping review and proposed roadmap. Healthc Manage Forum.

[91] Evans J., Leggat S.G., Samson D. (2023). A systematic review of the evidence of how hospitals capture financial benefits of process improvement and the impact on hospital financial performance. BMC Health Serv Res.

[92] Ard JL Jr, Tobin K., Huncke T., Kline R., Ryan S.M., Bell C. (2016). A Survey of the American Society of Anesthesiologists regarding environmental attitudes, knowledge, and organization. A A Case Rep.

[93] Petre M.A., Bahrey L., Levine M., van Rensburg A., Crawford M., Matava C. (2019). A national survey on attitudes and barriers on recycling and environmental sustainability efforts among Canadian anesthesiologists: an opportunity for knowledge translation. Can J Anaesth.

[94] Santos P., Oliveira B., Romão C., Leiria N. (2024). A survey on environmental sustainability among anesthesiologists: an opportunity for changing behaviors. Cureus.

[95] Kumar P, Sahani J, Rawat N, *et al.* Using empirical science education in schools to improve climate change literacy. *Renew Sustain Energy Rev* 023; 178: 113232.

[96] Dechezleprêtre A., Fabre A., Kruse T., Planterose B., Chico A., Stantcheva S. (2025). Fighting climate change: international attitudes toward climate policies. Am Econ Rev.

[97] Abousoliman A.D., Ibrahim A.M., Abualruz H. (2024). Exploring the relationship between nursing students’ knowledge and attitudes towards climate change and their psychological distress: a cross-national investigation. BMC Nurs.

[98] Huang A., Cooke S.M., Garsden C., Behne C., Borkoles E. (2024). Transitioning to sustainable, climate-resilient healthcare: insights from a health service staff survey in Australia. BMC Health Serv Res.

[99] Kotcher J., Maibach E., Miller J. (2021). Views of health professionals on climate change and health: a multinational survey study. Lancet Planet Health.

[100] NHS England (2025). The carbon literacy project. https://www.e-lfh.org.uk/programmes/carbon-literacy-for-healthcare/.

[101] Salas R.N., Maibach E., Pencheon D., Watts N., Frumkin H. (2020). A pathway to net zero emissions for healthcare. BMJ.

[102] Wyssusek K.H., Keys M.T., van Zundert A.A.J. (2019). Operating room greening initiatives - the old, the new, and the way forward: a narrative review. Waste Manag Res.

[103] Mazzei H.G., Specchia S. (2023). Latest insights on technologies for the treatment of solid medical waste: a review. J Environ Chem Eng.

[104] Hancher-Rauch H.L., Gebru Y., Carson A. (2019). Health advocacy for busy professionals: effective advocacy with little time. Health Promot Pract.

[105] Jasper J., Han Hahrie (2016).

[106] Iqbal S., Karia A., Kamming D., Herron D., O’Shea L., Vindrola-Padros C. (2024). Anaesthesia and climate change: time to wake up? A rapid qualitative appraisal exploring the views of anaesthetic practitioners regarding the transition to TIVA and the reduction of desflurane. BMC Anesthesiol.

[107] Gohad R., Jain S. (2024). Regional anaesthesia, contemporary techniques, and associated advancements: a narrative review. Cureus.

[108] McGain F., White S., Mossenson S., Kayak E., Story D. (2012). A survey of anesthesiologists' views of operating room recycling. Anesth Analg.

[109] Atcheson C.L., Spivack J., Williams R., Bryson E.O. (2016). Preventable drug waste among anesthesia providers: opportunities for efficiency. J Clin Anesth.

[110] Chaudhary K., Garg R., Bhalotra A.R., Anand R., Girdhar K. (2012). Anesthetic drug wastage in the operation room: a cause for concern. J Anaesthesiol Clin Pharmacol.

